# Fecal calprotectin: A promising readily available tool associated with outcome in patients with cirrhosis

**DOI:** 10.1002/ueg2.12640

**Published:** 2024-11-01

**Authors:** Florent Artru

**Affiliations:** ^1^ Liver Department Rennes University Hospital Rennes Bretagne France; ^2^ NuMeCan Institute Inserm U1241 Rennes Liver Failure Group (RELIEF) University of Rennes Rennes Bretagne France

**Keywords:** acute‐on‐chronic liver failure, bacterial translocation, Cirrhosis, inflammation

By traditional textbook knowledge, the development of variceal bleeding, hepatic encephalopathy, jaundice, and ascites marks the transition from compensated to decompensated cirrhosis. Decompensation commonly occurs in patients with clinically significant portal hypertension.^1^ Recent analyses, which have considered death as a competing event, have underscored the importance of the first decompensating event. These analyses highlighted that death is rarely observed in the absence of decompensation in patients with cirrhosis.^2^ Therefore, the long‐term mortality risk for patients who remain compensated is significantly lower compared to those who experience decompensation, with mortality rates being less than 15% and over 90% at 20 years, respectively.^2^

Once decompensation has occurred, the development of acute‐on‐chronic liver failure (ACLF), characterized by extra‐hepatic organ failure, becomes a major driver of mortality.^3^ It is estimated that this syndrome is encountered in about one‐third of patients admitted with acute decompensation (AD) of cirrhosis. The remaining two‐thirds of patients fall under the category of non‐ACLF decompensation, for which the best scoring systems associated with survival are MELD‐based ones and the Chronic Liver Failure Consortium AD score.^4^ These systems mainly rely on markers of liver disease dysfunction and renal dysfunction.

The PREDICT study, along with confirmation from external cohorts, has unveiled three distinct clinical courses of the disease.^5^ It is critical to identify those at the highest risk of developing ACLF (pre‐ACLF) in the short term. Identifying this population is of major interest, as they are the best candidates for the implementation of potential disease‐modifying agents to prevent the development of ACLF.

While the CLIF‐C AD score has been suggested to be helpful for this purpose it does not recapitulate accurately both severity of systemic inflammation and portal hypertension, the main drivers of liver‐related events and mortality in this population. Two important studies based on prospective cohorts have highlighted the significance of assessing markers of inflammation in addition to liver disease severity.^6^, ^7^ The markers identified were C‐reactive protein (CRP) and Presepsin (PSP). PSP is released from CD14 during inflammatory responses, where CD14 and its co‐receptor toll‐like receptor 4 on macrophages and monocytes recognize bacterial endotoxin. This specificity likely makes PSP a more accurate predictor of ACLF compared to the hepatically produced acute‐phase protein CRP. Consequently, in the latest model development (Padua Model 2.0), PSP was shown to be superior to CRP in predicting the occurrence of ACLF.^7^ However, PSP currently lacks immediate clinical applicability as it is not available in most centers managing patients with AD.

In the present study, Koller et al. explored the potential of fecal calprotectin (F‐CAL) to predict adverse outcomes (liver transplantation and death) in a large prospective cohort of hospitalized patients with advanced chronic liver disease (ACLD).^8^ F‐CAL is a sensitive marker of intestinal inflammation, commonly used for screening and monitoring intestinal inflammation in the context of chronic diarrhea and inflammatory bowel disease. F‐CAL has the advantage of being available worldwide with standardized methods of measurement. Preliminary studies suggested that calprotectin either in ascites, plasma or in feces could be associated with intestinal inflammation in the context of ACLD as well.^9^, ^10^ This association may illustrate a degree of disruption of the intestinal barrier and bacterial translocation, major contributors to systemic inflammation and the worsening of PHT.^8^ The authors specifically observed that F‐CAL was an independent predictor of adverse outcomes, with predictive ability directly correlating with F‐CAL values. Importantly, this predictive ability was enhanced in subgroups of patients with less severe disease, who theoretically have the highest chance of benefiting from disease‐modifying agents and could expect long‐term stabilization or recompensation. A high fecal F‐CAL value greater than 11 times the upper limit of normal, observed in more than 25% of patients, was associated with the highest risk of adverse outcomes, especially in patients with a CLIF‐C AD score below 50. Additionally, the authors explored the association of F‐CAL values with patient phenotypes and circulating markers of inflammation. Despite acknowledged limitations (including especially the relationship between lactulose, rifaximin or beta‐blockers therapy and F‐CAL values) and the need for a validation study, the present study paves the way for the use of F‐CAL measurement, a readily and widely available tool, in patients with AD to help identify those who could benefit from early intervention to reverse the natural course of the disease.

## CONFLICT OF INTEREST STATEMENT

The author have no conflicts of interest to declare.
